# Efficiently recording and processing data from arbuscular mycorrhizal colonization assays using AMScorer and AMReader

**DOI:** 10.3389/fpls.2024.1405598

**Published:** 2024-05-17

**Authors:** Edwin Jarratt-Barnham, Giles E. D. Oldroyd, Jeongmin Choi

**Affiliations:** Crop Science Centre, University of Cambridge, Lawrence Weaver Road, Cambridge, United Kingdom

**Keywords:** AMReader, AMScorer, arbuscular mycorrhizal fungi, microscopy, root length colonization

## Abstract

Arbuscular mycorrhizal (AM) fungi engage with land plants in a widespread, mutualistic endosymbiosis which provides their hosts with increased access to nutrients and enhanced biotic and abiotic stress resistance. The potential for reducing fertiliser use and improving crop resilience has resulted in rapidly increasing scientific interest. Microscopic quantification of the level of AM colonization is of fundamental importance to this research, however the methods for recording and processing these data are time-consuming and tedious. In order to streamline these processes, we have developed AMScorer, an easy-to-use Excel spreadsheet, which enables the user to record data rapidly during from microscopy-based assays, and instantly performs the subsequent data processing steps. In our hands, AMScorer has more than halved the time required for data collection compared to paper-based methods. Subsequently, we developed AMReader, a user-friendly R package, which enables easy visualization and statistical analyses of data from AMScorer. These tools require only limited skills in Excel and R, and can accelerate research into AM symbioses, help researchers with variable resources to conduct research, and facilitate the storage and sharing of data from AM colonization assays. They are available for download at https://github.com/EJarrattBarnham/AMReader, along with an extensive user manual.

## Introduction

**Figure 1:**
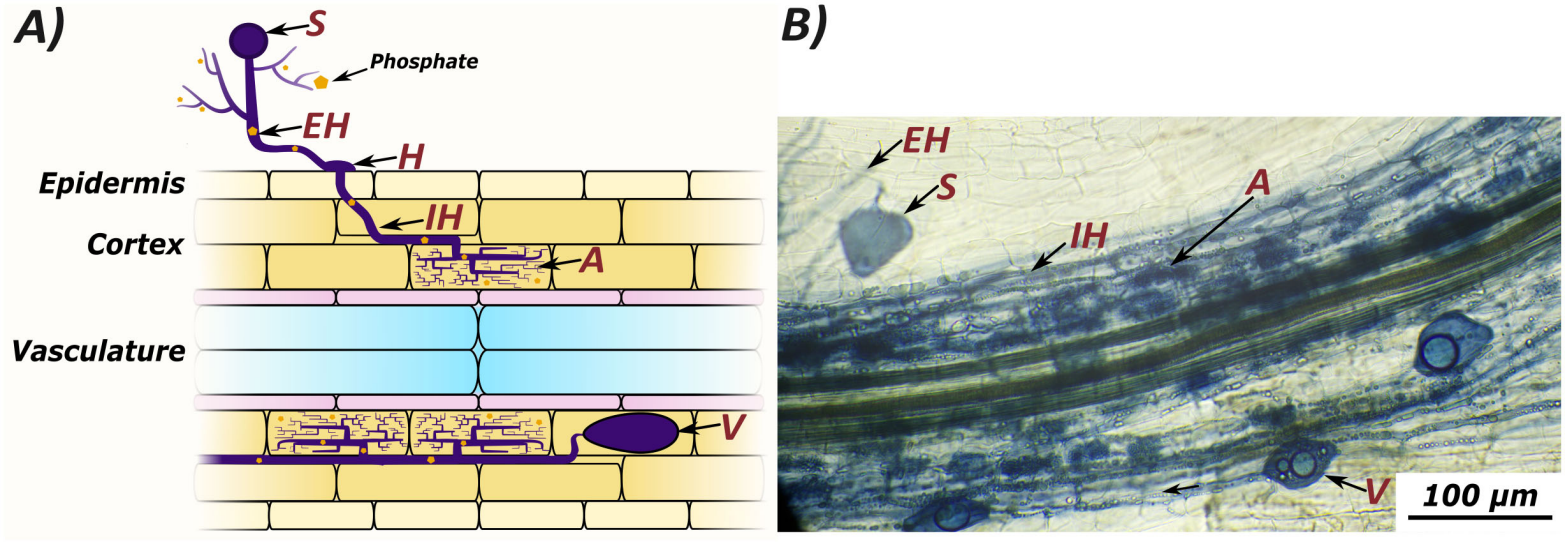
Arbuscular mycorrhizal colonization can be quantified by counting several microscopically visible structures: Spores (S), extraradical hyphae (EH), hyphopodia (H), intraradical hyphae (IH), arbuscules (A), and vesicles (V). Extraradical hyphae scavenge nutrients from the growth medium, which are transferred to the host plant at arbuscules. A graphic representation of fungal structures **(A)** compared to an image of colonised root tissue from *Lotus japonicus* stained with ink and vinegar **(B)**.

The evolution of land plants has been shaped by their interactions with a diverse microbial community. Amongst these interactions, the mutually beneficial symbiosis with arbuscular mycorrhizal (AM) fungi is one of the oldest and most widespread (Remy et al., 1994; Treseder and Cross, 2006). At the start of this symbiosis, fungal spores germinate and extend extraradical hyphae. Then, upon contact with a potential host, AM fungi develop hyphopodia on the surface of epidermal cells, enter the root, and produce intraradical hyphae. Subsequently, AM fungi develop arbuscules in the cortex, and establish vesicles and new spores (**Figure 1**) (Smith and Read, 2008; van Creij et al., 2020). Arbuscules are the characteristic, highly branched structures which AM fungi produce beneath the walls of plant cells, surrounded by a plant-derived membrane. Across this membrane, plants provide the fungus with sugars and fatty acids, in return for inorganic nutrients such as phosphorous and nitrogen (Luginbuehl and Oldroyd, 2017; Paries and Gutjahr, 2023). In vascular plants, fungal colonization is restricted to root tissue, while in non-vascular plants AM fungi colonise other tissues, such as the thalli of liverworts and hornworts (Edwards et al., 2000; Wang and Qiu, 2006).

AM fungi and their host plants are globally distributed (Treseder and Cross, 2006; Öpik et al., 2010), and AM fungi play important roles in both natural ecosystems and agricultural environments, offering plants both improved access to nutrients and increased abiotic and biotic stress resistance (Begum et al., 2019; Samuel and Veeramani, 2021; Bennett and Groten, 2022). Given their ecological importance, their significance for plant biology, and the potential for agricultural benefit, AM fungi have received increasing scientific and industrial interest.

Most research into AM symbioses requires a means of assessing the abundance of AM fungi within the roots and rhizosphere. The earliest studies of AM fungi were dependent on microscopic analysis of stained plant tissue. While many alternative methods for quantifying AM colonization have since been developed using molecular proxies for the presence of AM fungi (**Table 1**), microscopy remains the gold-standard. This is because microscopy provides a direct measurement of fungal abundance without the need for homogenization of plant tissue. Consequently, microscopy enables the simultaneous assessment of the abundance, spatial distribution, and morphology of specific fungal structures. Microscopy, however, can be extremely time-intensive, and so represents a bottleneck for research into AM symbioses. Therefore, ways to efficiently record and process data from microscopic observations is of significant value.

**Table 1:** Methods to quantify AM colonization.

Type	Method	Details	References
Nucleic acids			
Fungal DNA	DNA amplicon sequencing	• Sequencing of the internal transcribed spacer sequence (ITS). • Quantification and identification of fungal diversity. • Commonly used in ecological and field-based studies. • Absolute quantification may be challenging (Alteio et al., 2021).	(Schoch et al., 2012; Tedersoo et al., 2015; Kryukov et al., 2020; Alteio et al., 2021)
	qPCR	• Targeted quantification of DNA using species-specific primers. • High-throughput. • Measures relative fungal abundance. • Fungal spores have more effect on the final measurement compared with fungal hyphae (Gamper et al., 2008).	(Gamper et al., 2008; Voříšková et al., 2017; Bodenhausen et al., 2021)
Fungal RNA	qRT-PCR	• Fungal housekeeping genes provide the abundance of living fungus (e.g. *Rhizophagus irregularis elongation factor 1α*). • Requires well-characterised marker genes for each fungal species. • Correlations between the microscopic assessment and multiple fungal gene expressions have been assessed in grapevine (Duret et al., 2022).	(Pérez-Tienda et al., 2014; Choi et al., 2020; Evangelisti et al., 2021; Duret et al., 2022; Shi et al., 2022)
Plant RNA	qRT-PCR	• AM symbiosis-specific gene expression. • Requires well-characterised marker genes. • Employed alongside microscopic methods to assess the molecular functioning of AM symbioses. • Abundance of specific plant transcripts may not correlate with microscopic assessment depending on the symbiotic state of the plant (Isayenkov et al., 2004).	(Isayenkov et al., 2004; Gutjahr et al., 2008; Fiorilli et al., 2009; Gomez et al., 2009; Gaude et al., 2012; Hogekamp and Küster, 2013; Shi et al., 2021; Das et al., 2022)
Metabolites			
Plant pigments naturally accumulating during AM symbiosis	Visible	• Mycorradicin. • A yellow, carotenoid-derived pigment. • Plant-specific (e.g. maize). • Rapid and easy. • Well-suited to genetic screening.	(Klingner et al., 1995)
	Visible	• Red-brown pigment. • Plant-specific (e.g. *Marchantia paleacea*). • The identity of this pigment is unknown.	(Hata et al., 2010; Kodama et al., 2022)
Plant pigment accumulating due to a genetically engineered reporter system	Visible	• Betalain or anthocyanin. • Easy and rapid. • Well-suited to genetic screening. • Requires transgenic plants. • Irreversible accumulation represents cumulative, rather than dynamic, colonization.	(Timoneda et al., 2021; Kumar et al., 2022)
Fungal metabolites	GC/MS	• Fatty acids (e.g. neutral lipid fatty acid 16:1ω5 and phospholipid fatty acid 16:1ω5). • May not be specific to AM fungi (Ngosong et al., 2012).	(Olsson et al., 1995, Olsson et al., 1998; Ngosong et al., 2012)
Plant metabolites	LC/MS	• Blumenol in leaf tissue. • No root sampling. • High-throughput. • No transgenic plants required. • Applies to many plant species.	(Wang et al., 2018)

qPCR, quantitative polymerase chain reaction; qRT-PCR, quantitative reverse transcription-polymerase chain reaction; GC/MS, Gas chromatography/mass spectrometry; LC/MS, Liquid chromatography/mass spectrometry.

To ease data collection by microscopic techniques, we have developed an Excel spreadsheet (AMScorer) which enables a user to easily record the presence or absence of all six fungal structures (extraradical hyphae, hyphopodia, intraradical hyphae, arbuscules, vesicles, and spores) during microscopic assays. An accompanying R package, AMReader, can read the data in AMScorer to rapidly produce graphical outputs with statistical analyses. These tools are user-friendly, compatible with many established methods of quantifying AM fungi, and are available at https://github.com/EJarrattBarnham/AMReader along with a comprehensive manual. Here, we present their key features.

## Method

**Figure 2:**
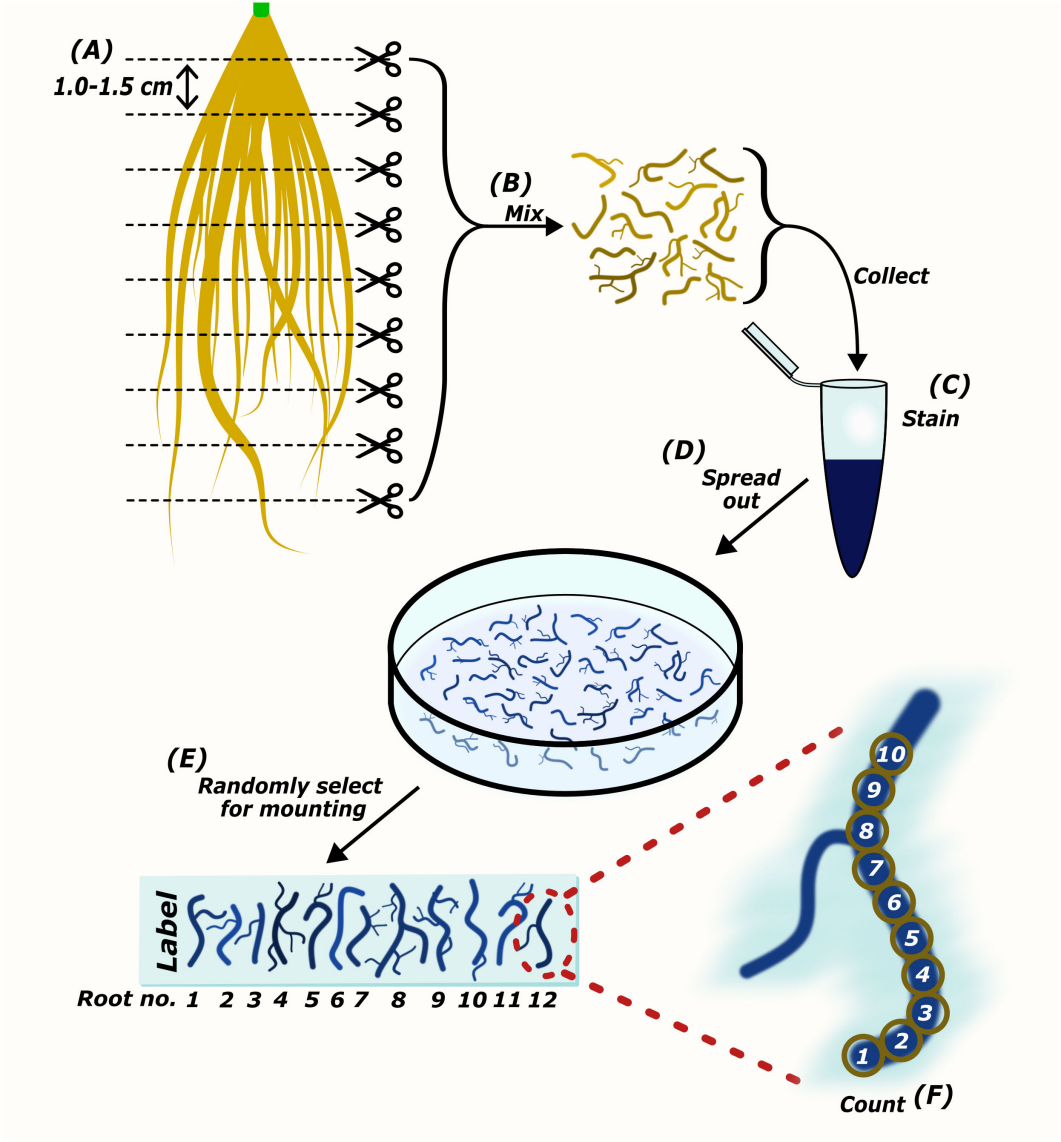
The preparation of root samples for microscopic quantification of AM colonization. Harvested roots are cut into sections approximately 1.0-1.5 cm in length **(A)**. Root pieces are mixed thoroughly to collect representatives of the whole root system for staining **(B, C)**. Root pieces are spread out in mounting medium **(D)**. Randomly selected root pieces are mounted onto a microscopy slide **(E)**. The abundance of AM fungi is counted either per field-of-view (Torabi et al., 2021), or using the McGonigle magnified intersection method (McGonigle et al., 1990) **(F)**.

AMScorer has been developed to aid the recording and processing of data from AM root colonization assays. It is paired with AMReader to enable quick statistical analysis and data visualization. They are primarily designed to be compatible with the McGonigle magnified intersection method (McGonigle et al., 1990), or modifications thereof (Torabi et al., 2021). They can also be utilized for methods which quantify AM colonization in similar ways, such as the gridline intersect method (Giovannetti and Mosse, 1980). The specific details of these methods have been reviewed (Ho-Plágaro et al., 2020; Torabi et al., 2021). An example workflow for preparation of this material is outlined in **Figure 2**. Following preparation of the root material, AMScorer can be used to record AM colonization levels in either 100 fields-of-view or 100 intersections, recording the presence/absence of each fungal structure. For large root systems, it is critical to collect representative root fragments by thoroughly randomizing their selection. It is also possible to quantify multiple slides of root fragments, and subsequently average the results.

### AMScorer

**Figure 3:**
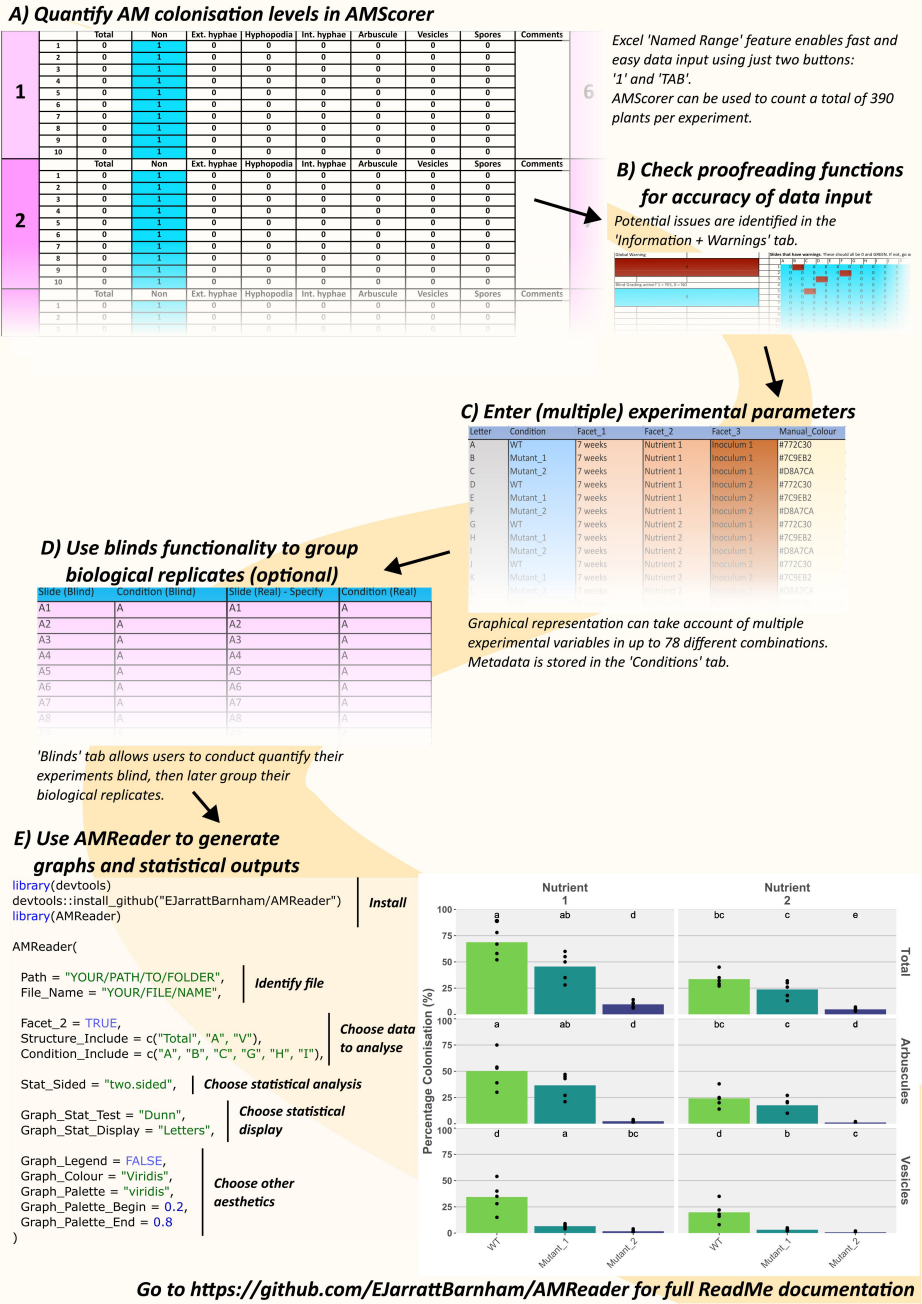
An example workflow using AMScorer and AMReader. One ‘Counting Table’ per plant allows the quantification of colonization in 390 plants per experiment **(A)**. Proofreading methods ensure accurate data input **(B)**. The ‘Conditions’ sheet can account for multiple experimental parameters with up to 78 different combinations **(C)**. AMScorer enables easy blind counting and data to be grouped in multiple ways **(D)**. An example R code to read AMScorer sheet into AMReader **(E)**. AMReader provides an easy way to (1) read data from AMScorer, (2) select which data to present, (3) conduct various statistical analyses, (4) display data alongside statistical information, and (5) adjust color schemes and other aesthetics of graphical outputs.

**Figure 4:**
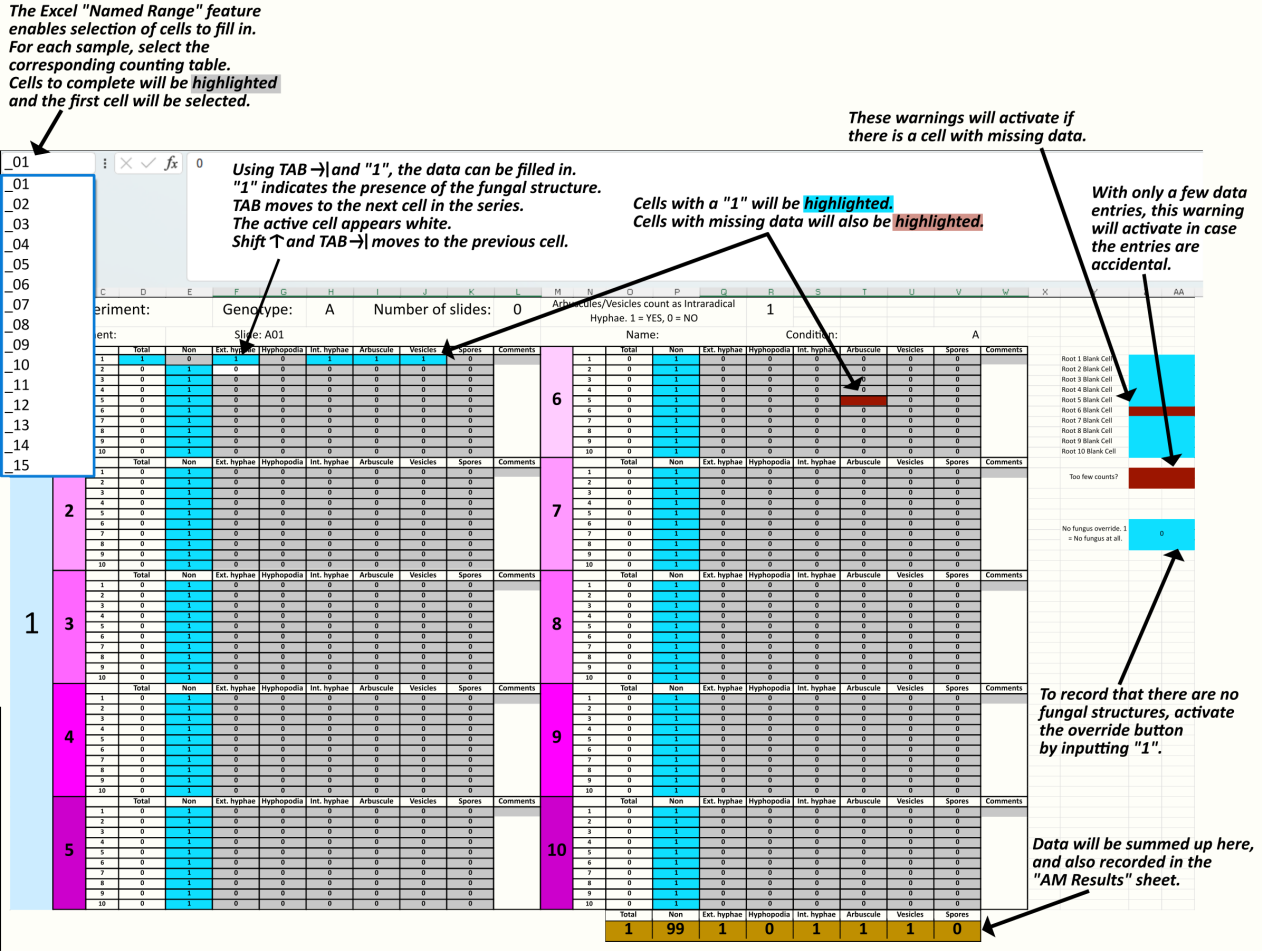
The main features of each counting table in AMScorer. The Excel “Named Range” feature enables selection of cells for data entry. Movement to the next cell in the counting table is achieved with the keyboard TAB button. Pressing SHIFT and TAB will allow movement to the previous cell. Cells where the presence of a fungal structure have been recorded change color to blue, whilst cells where there is no data entered will turn red and activate a corresponding warning. If AMScorer believes a data entry may have been accidental, it will highlight the “Too few counts?” warning. To record that no fungal structures were present in a sample, activate the “No fungus override” button by inputting a “1”. Counts of fungal structures are summed up below each table, and results transferred automatically to the “AM Results” sheet.

AMScorer (Excel) consists of 26 sheets (A to Z), each of which contains 15 counting tables (**Figure 3A**) (**Supplementary File 1**). Each table is intended to collect data from a biological replicate, often a single plant. In total, AMScorer can collect data from 390 plants per experiment, which we expect to be sufficient for large experimental designs. The counting tables exploit the Excel ‘Named Range’ sheet feature to enable all data inputs using just two buttons: ‘1’ and ‘TAB’ (**Figure 4**). This allows the user to operate the spreadsheet with one hand, while adjusting the microscope’s field-of-view with their other hand. Consequently, data can be collected simultaneously to microscopic observations. This greatly reduces the time needed for data collection. The data from these counting tables are then automatically fed into the main output, the ‘AM Results’ sheet, which displays percentage root length colonization of each structure per replicate. An ‘Information + Warnings’ sheet collects information about possible errors in data input to help ensure data accuracy (**Figure 3B**).

AMScorer also contains a ‘Conditions’ sheet where the user can provide metadata detailing the experimental parameters (**Figure 3C**). This may include, for example, genotypes, time points, and growth conditions. AMScorer can handle complex experimental designs with up to 4 different variables, and a total of 78 unique combinations of these variables.

Additionally, a ‘Blinds’ sheet enables users to perform colonization assays blind, and for collected data to be readily reorganized into sets of biological replicates (**Figure 3D**). A ‘Test’ sheet contains 15 counting tables which may be used for collecting data that is not intended to contribute to the main results, but which may be useful to the user. For example, it may be used to keep a record of colonization from plants sampled during an experiment to inform the timing of a full harvest.

Through these features, AMScorer enables rapid and easy collection of data, which can subsequently be maintained and shared alongside information concerning the experimental design. Full details on the use of AMScorer are detailed in the documentation found in the GitHub repository.

### AMReader

**Figure 5:**
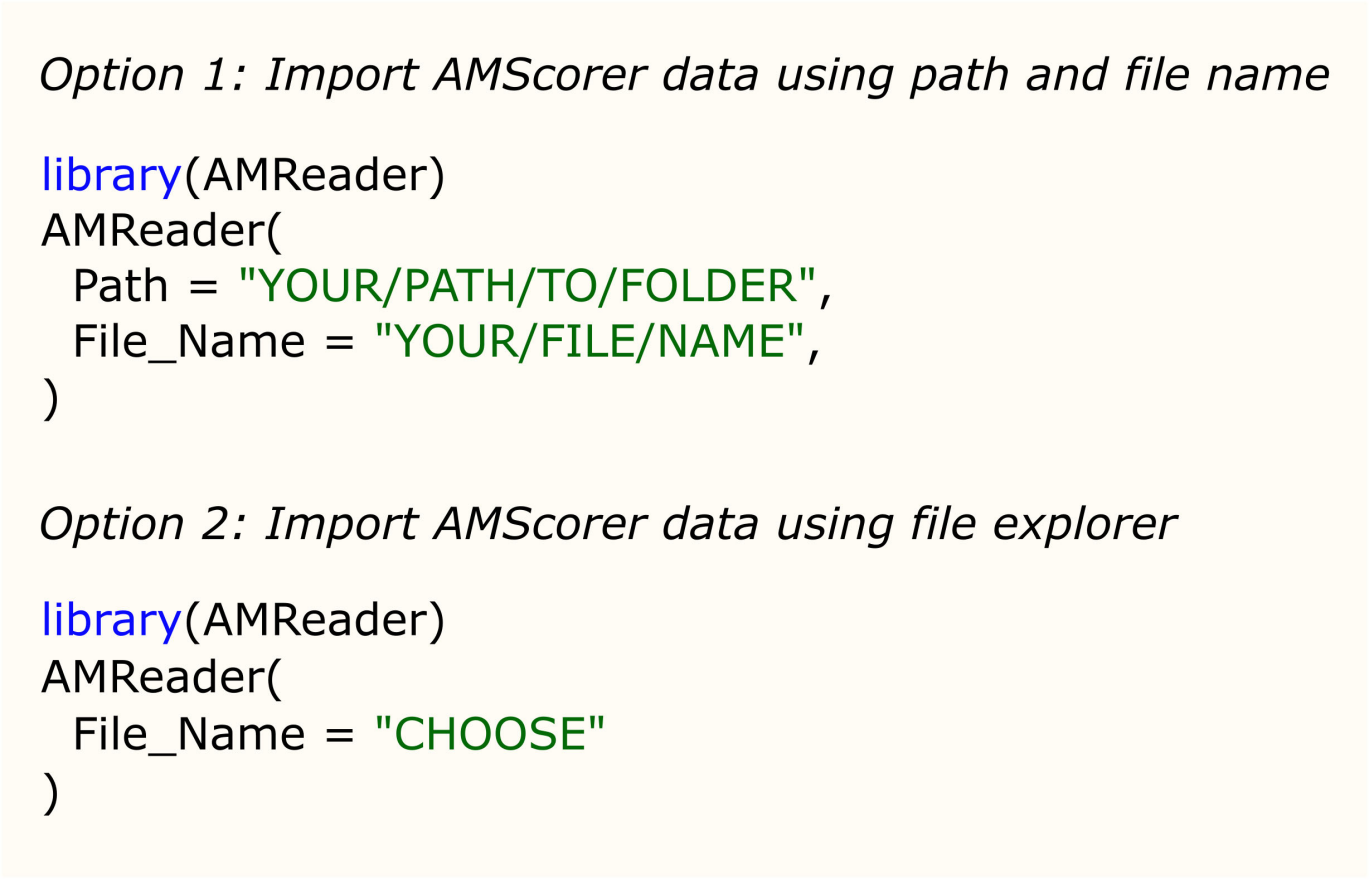
The two options for importing data from AMScorer into AMReader.

Following data collection, AMScorer can be read by the R package AMReader, which has been designed to: (1) Import data from AMScorer and process these data, (2) conduct statistical analyses, and (3) generate graphical displays (**Figure 3E**). Requiring only basic skills in R software, AMReader enables rapid and simple data analysis with just a single function. Its simplest input requires only the identification of the AMScorer file. This can be achieved either by defining its path and file name, or opening file explorer to locate the file (**Figure 5**). AMReader also contains extensive options for selecting data, choosing statistical tests, and adjusting the aesthetics of graphical outputs (**Table 2**). All these operations are controlled by the AMReader function, creating a user-friendly method to personalize and edit the output. A full set of examples, working through an example experimental dataset, are detailed in the documentation on the GitHub repository. For more experienced R users, the key outputs of AMReader are saved to the R environment, enabling user customization at any stage.

**Table 2:** A list of variables available in AMReader.

Stage	Purpose		Variable(s)
Data input*	Identify file to analyse*		Path*
			File_Name*
	Data to analyse		Condition_Include
			Condition_Exclude
			Structure_Include
			Structure_Exclude
	Experimental variables to process		Facet_1, Facet_2, Facet_3
Statistical Analysis	p-value adjustment method		Stat_Dunn_Padj
			Stat_Wilcoxon_Padj
	One- or two-sided statistical tests		Stat_Sided
	Save statistics		Stat_Output
	Name of statistics output		Stat_File
Graph Production	Graph style		Graph_Type
	Bars or Boxes		Graph_Object
	Include individual datapoints		Graph_Datapoints
	Bar order	Order of each condition Order of additional experimental variables (facets)	Graph_Condition_Order Graph_Facet_1_Order, Graph_Facet_2_Order, Graph_Facet_3_Order
	Statistical information	Display results from statistical tests Display compact letter groups or use asterisks Reference condition if displaying asterisks	Graph_Stat_Test Graph_Stat_Display Graph_Reference_Condition
	Bar colours	Metadata from AMScorer R color set to use R color palette to use from color set Start point for continuous color palettes End point for continuous color palettes	Graph_Manual_Colour Graph_Colour Graph_Palette Graph_Palette_Begin Graph_Palette_End
	Other colours	Text color Background color Colour of horizontal lines	Graph_Text_Colour Graph_Background_Colour Graph_Hline_Colour
	Number of biological replicates		Graph_Sample_Sizes
	Figure legend		Graph_Legend
	Graph size	Label size on the right-hand side of the graph Label size on the top of the graph Y-axis text size X-axis text size Figure legend size Figure legend text size Y-axis percentage size Statistical information size Datapoint size	Graph_Size_Right_Label Graph_Size_Top_Label Graph_Size_Y_Axis Graph_Size_X_Axis Graph_Size_Legend Graph_Size_Legend_Text Graph_Size_Percentages Graph_Size_Statistics Graph_Size_Datapoints
	Save graphical output		Graph_Output
	Graph resolution		Graph_Resolution
	Width of the saved graph		Graph_Width_Adjustment
	Height of the saved graph		Graph_Height_Adjustment
	Name of graphical output		Graph_File

*All variables, except ‘Path’ and ‘File_Name’, are optional, with reasonable defaults chosen in each case. See documentation for more information on each variable, and examples of their use.

## Results

**Figure 6:**
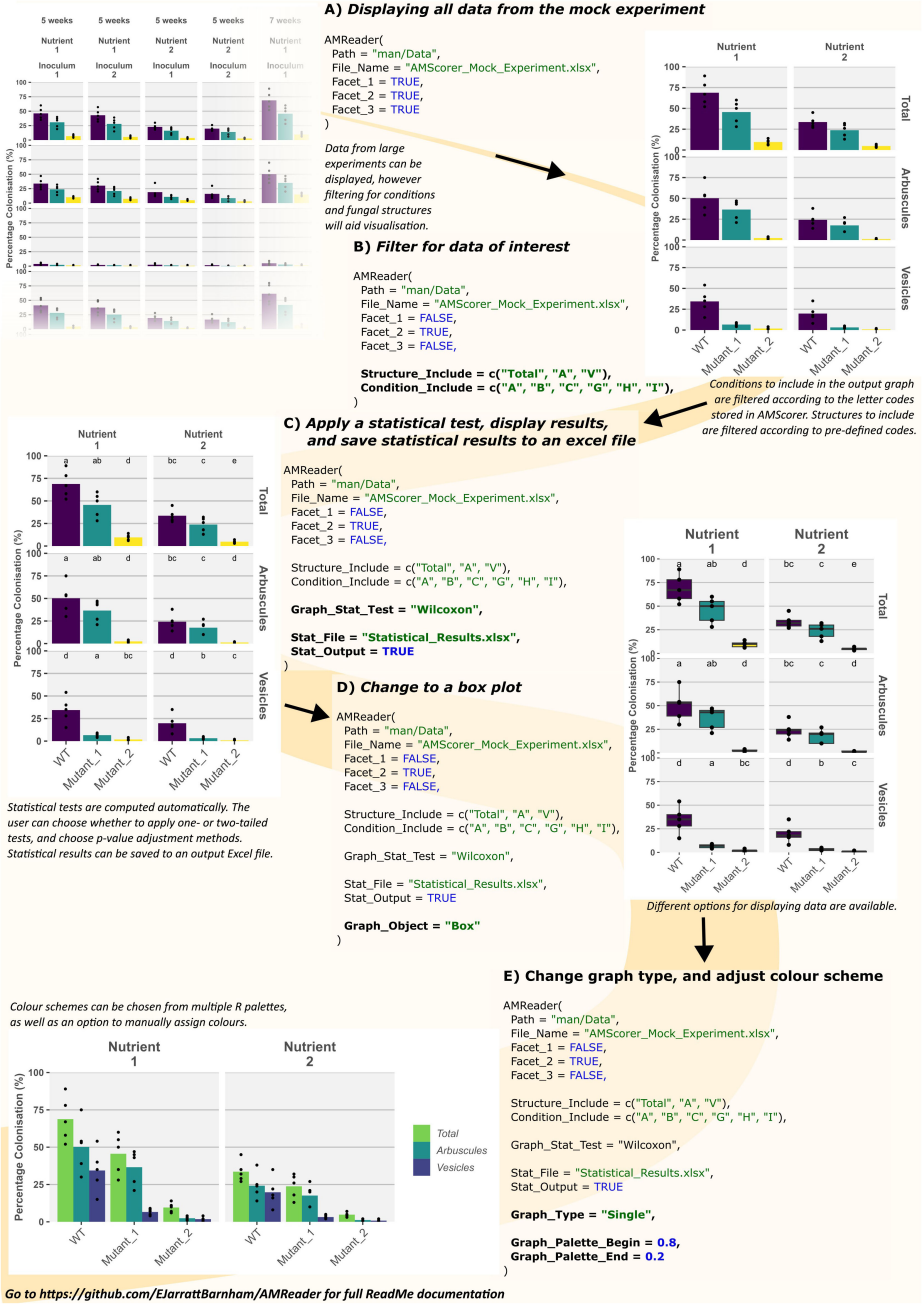
Example usage of AMReader. A simple workflow would involve loading data by identification of the AMScorer file **(A)**, filtering of data to choose only those conditions and fungal structures of interest **(B)**, statistical analysis **(C)**, and later aesthetic changes to the graphical representation **(D, E)**.

Once microscopic quantification has been performed using AMScorer, the data will be automatically collected within the ‘AM Results’ sheet. In order to demonstrate the downstream functions of AMReader, a simulated dataset was generated (**Supplementary File 2**). This dataset is accessible within the GitHub repository under ‘man/Data’. **Figure 6** demonstrates an example workflow for data analysis, which is also described below.

Firstly, the experimental data was loaded and displayed in full by activating all three faceting variables, corresponding to the three different experimental variables contained in the simulated data (**Figure 6A**). Subsequently, the data was filtered using the ‘Structure_Include’ and ‘Condition_Include’ variables (**Figure 6B**). Conditions were selected according to the letter codes stored in the ‘Conditions’ sheet (**Figure 3C**), and structures were selected according to their own codes (**Table 3**). Subsequently, statistical analysis was conducted by defining the statistical test using the ‘Graph_Stat_Test’ variable (**Figure 6C**). The user can choose from a range of parametric and non-parametric statistical tests, including the TukeyHSD and Wilcoxon pairwise tests. Using ‘Stat_Output’ and Stat_File’ would also allow the user to save an excel file containing reports from any statistical tests conducted on the given data.

**Table 3:** Codes for filtering structures within AMReader.

Structure	Method
Total colonization	Total
Extraradical hyphae	EH
Intraradical hyphae	IH
Arbuscules	A
Vesicles	V
Spores	S

AMReader currently supports multiple ways of displaying colonization data. For instance, a box plot may be preferable to bar plots, and this option can be chosen using the Graph_Object variable (**Figure 6D**). In addition, it is possible to produce a graph where the quantification of fungal structures is either separated into distinct facets (Graph_Type = “Facets”), or displayed alongside each other (Graph_Type = “Single”) (**Figure 6E**). Further personalization of the graph can be achieved by a great range of variables (**Table 2**), as illustrated here by changing the color of bars (**Figure 6E**).

We will seek to expand the range of graphical representations available in the near future, and would also welcome contributions from the community. Alternatively, all datafiles generated during the operation of AMReader are saved to the R environment so that more experienced R users can use AMReader for initial analyses, and then generate figures and results according to their own preferences.

## Discussion

Quantification of AM colonization by microscopy represents a significant bottleneck for the study of AM symbioses. Many alternative, high-throughput methods have been developed to quantify AM colonization through molecular and metabolic changes induced by AM colonization. However, these rarely capture information on the spatial distribution, relative abundance, and morphological features of fungal structures. Consequently, microscopy remains a key requirement for many studies into AM symbioses. To reduce the time required for AM colonization assays, we have developed an Excel spreadsheet, AMScorer, which enables the user to record data during microscopy-based assays, and instantly performs the subsequent data processing steps. Over the course of an experiment, this greatly reduces the time required for quantification of AM colonization. In addition, AMScorer enables all data relevant to the experiment to be stored within a single spreadsheet, allowing easier preservation and dissemination of AM colonization data.

Recently, a novel machine learning approach has also been developed to aid the collection of AM colonization data (Evangelisti et al., 2021). Such methods hold promise for enabling full automation of AM colonization assays. Currently, however, they require high-resolution images with good contrast, which can be difficult to obtain in some plant species and are also time-intensive to acquire. Additionally, these tools require significant expertise in the computational methods. Here we provide tools which require limited technical knowledge, and are compatible with currently established methods for conducting colonization assays, and are compatible with downstream analysis pipelines, such as Ramf (Chiapello et al., 2019).

To facilitate data analysis, we have also developed AMReader, an R package which can transform the data collected by AMScorer into graphical representations quickly and easily. We have sought to develop AMReader so that it is usable by those with little experience in R software, creating a user-friendly system in which the user can control the R pipeline, without the need to write any additional layers of code. This distinguishes AMReader from currently available pipelines. We have, however, also ensured that more experienced R users can employ AMScorer and AMReader within their own, personalized, scripts.

Microscopic quantification of AM colonization represents a major bottleneck for research into AM colonization. AMScorer and AMReader represent new tools which can more than halve the time necessary for assays. We hope this will greatly accelerate research and facilitate sharing of data within the community.

## Data availability statement

The datasets presented in this study can be found in online repositories. The names of the repository/repositories and accession number(s) can be found in the article/**Supplementary Material**.

## Author contributions

EJ-B: Conceptualization, Software, Validation, Writing – original draft, Writing – review & editing. GO: Funding acquisition, Writing – review & editing, Writing – original draft. JC: Funding acquisition, Writing – original draft, Writing – review & editing.
